# Group Qigong for Adolescent Inpatients with Anorexia Nervosa: Incentives and Barriers

**DOI:** 10.1371/journal.pone.0170885

**Published:** 2017-02-02

**Authors:** Juliette Gueguen, Marie-Aude Piot, Massimiliano Orri, Andrea Gutierre, Jocelyne Le Moan, Sylvie Berthoz, Bruno Falissard, Nathalie Godart

**Affiliations:** 1 Université Paris-Saclay, Univ. Paris-Sud, UVSQ, CESP, INSERM, Paris, France; 2 Univ. Paris-Descartes, Paris, France; 3 Psychiatry Unit, Institut Mutualiste Montsouris, Paris, France; TNO, NETHERLANDS

## Abstract

**Background:**

Qigong is a mind-body intervention focusing on interoceptive awareness that appears to be a promising approach in anorexia nervosa (AN). In 2008, as part of our multidimensional treatment program for adolescent inpatients with AN, we began a weekly qigong workshop that turned out to be popular among our adolescent patients. Moreover psychiatrists perceived clinical benefits that deserved further exploration.

**Methods and findings:**

A qualitative study therefore sought to obtain a deeper understanding of how young patients with severe AN experience qigong and to determine the incentives and barriers to adherence to qigong, to understanding its meaning, and to applying it in other contexts. Data were collected through 16 individual semi-structured face-to-face interviews and analyzed with the interpretative phenomenological analysis method. Eleven themes emerged from the analysis, categorized in 3 superordinate themes describing the incentives and barriers related to the patients themselves (individual dimension), to others (relational dimension), and to the setting (organizational dimension). Individual dimensions associated with AN (such as excessive exercise and mind-body cleavage) may curb adherence, whereas relational and organizational dimensions appear to provide incentives to join the activity in the first place but may also limit its post-discharge continuation. Once barriers are overcome, patients reported positive effects: satisfaction associated with relaxation and with the experience of mind-body integration.

**Conclusions:**

Qigong appears to be an interesting therapeutic tool that may potentiate psychotherapy and contribute to the recovery process of patients with AN. Further analysis of the best time window for initiating qigong and of its place in overall management might help to overcome some of the barriers, limit the risks, and maximize its benefits.

## Introduction

Mind-body interventions, which encompass a large range of approaches, including qigong, tai chi, yoga, and meditation, combine body movements with mental focus [1]. They are practiced both to reduce psychological distress and boost well-being [1]. Because of their high potential for both the prevention [2] and management of anorexia nervosa (AN) [3], they have been progressively implemented in multidimensional treatment programs for eating disorders. Instructors of these techniques contend that they contribute to the recovery process in a wide variety of ways: through relaxation, enhancing body awareness and acceptance, and modifying perceptions of life events [4].

Qigong is a mind-body intervention and a branch of traditional Chinese medicine [5]. It involves slow movements, breathing, and meditation exercises. Qigong challenges the foundations of modern Western biomedical thought, sharing as it does the philosophy of traditional Chinese medicine and its aim to strengthen *qi* (life energy) throughout the body. Although scientific evidence of its efficacy is undeniably scarce, a recent meta-analysis suggests that qigong exercise reduces stress and anxiety in healthy adults [6]. In qualitative studies exploring its perceived benefits, older adults report body-mind-spirit integration as a main effect as well as physical, mental, emotional, social, and spiritual benefits [7], and participants’ sense of strong connection to both themselves and the other participants during group practice appear to produce positive psychosocial effects [8].

In 2008, our multidimensional treatment program [9–11] for adolescent inpatients with AN, in the adolescent and young adult psychiatric department of the Montsouris Medical Institute (IMM Hospital), began a weekly qigong workshop. AN is characterized by food restriction, severe weight loss, a distorted body image, and a pathological fear of becoming fat [12,13]. Prevalence estimates among adolescents from the most recent epidemiological studies range from 0.3% [14] and 0.5% [15] to 1.7% [16]. Intense physical activity, anxiety concerning weight and shape, and recurrent negative thoughts about their distorted body image are major features of AN. The association of severe AN with poor outcomes, including mortality and chronic morbidity [17], underlines the need to develop new approaches.

Qigong focuses on interoceptive processes (proprioception and visceroception), that is, awareness of stimuli that arise within the body. For that reason, it appears to be a promising approach in AN, whose features include difficulties in perceiving internal bodily signals [18], that can either be measured in an objective manner (interoceptive accuracy, measured with the heartbeat detection task [19]) or with psychometrics (interoceptive awareness, measured with the interoceptive awareness subscale of the Eating Disorder Inventories [20]).

Despite a careful search in Medline and PsychINFO, we have found no data about qigong in AN. A few studies have evaluated other mind-body therapies in eating disorders but both quantitative and qualitative data are scarce. Recently, Vancampfort et al. [21] reviewed the existing randomized controlled studies of the use of aerobic exercise, yoga, massage, and basic body awareness in the management of anorexia and bulimia nervosa, but the paucity and heterogeneity of the studies prevented them from reaching any firm conclusions. Among patients aged 11–21 years with eating disorders, including AN, yoga reduced preoccupation with food immediately after all sessions and significantly lowered EDE (eating disorders examination interview) scores after 12 weeks compared to control patients on a waiting list [22]. A qualitative study provided enlightening information about how the practice of yoga by women in a program for binge eating helped them move from a state of distraction and feeling physically absent (associated with binge eating) to a state of feeling focused and physically present (associated with satiation) [23]. Likewise, a study among patients with eating disorders of the Feldenkrais method, based on "awareness through movement", reported that it contributed to the development of "a felt sense of self, self-confidence and a general process of maturation of the whole personality" [24].

Because our program’s qigong workshop was popular among our adolescent patients, and psychiatrists perceived clinical benefits that deserved further exploration, we undertook this study. Our objective was to obtain a deeper understanding of how young patients with severe AN experience qigong and to determine the incentives and barriers to adherence to qigong, to understanding its meaning, and to applying it in other contexts. We also sought to ascertain its potential benefits and risks. We anticipated that this information could help clinicians to understand which patients might benefit most from the qigong program and would also provide useful insights into how best to supervise and integrate this program into each patient’s overall management to maximize its benefits and minimize its risks.

## Methods

### Participants and recruitment

Patients were recruited from January 1 to March 30, 2014 from eligible subjects hospitalized in the adolescent and young adult psychiatric department of IMM Hospital for AN or EDNOS (eating disorders not otherwise specified, i.e. diagnosis of AN missing 1 or 2 criteria), aged 13–21, with current or previous access to a qigong group session. To facilitate reading hereafter, we will refer to all patients as patients with AN. Qigong attendance was offered throughout the entire duration of hospitalization (which generally lasted several months), once patients were medically stable. Most patients participated voluntarily, attending regularly or irregularly, depending on their schedule (since it could conflict with their school programs) and their preference. If the physician in charge deemed it appropriate, the activity might have been strongly advised (prescribed) and therefore not dependent on the patient’s free choice.

Sampling was purposive [25] and inclusion continued until sufficient data were obtained [26]. Accordingly, we included patients as heterogeneous as possible, including those who attended qigong regularly and those who came irregularly. We were therefore able to consider a wide range of situations and experiences.

Finally, data from 16 young women were sufficient; because 4 refused to participate, we ended up asking 20; the 16 participants freely consented, as described below. Their median age was 17 years (13–19) at the interview. Qigong was prescribed for 1 adolescent; the remaining 15 reported that their attendance depended only on their schedule and personal choices. Ten had a history of regular attendance (see Table 1). Patients with irregular attendance will be referred as (irreg) in the quotations. Attendance could be irregular for various reasons, including low adhesion, refusal to participate, and concomitant activity (such as class). This number of subjects sufficed for in-depth analysis according to the methodology we used, Interpretive Phenomenological Analysis (IPA) [25,26], described below.

**Table 1:** Participants' characteristics.

Patient	Age (years)	BMI (kg/m^2^) (admission)	BMI (kg/m^2^) (interview)	Duration of AN (years)	Duration of hospitalization (months)	Self-reported Qigong attendance		Duration of interview (minutes)
A	17	12.8	19.5	2.5	7	Regular		21
B	13	16.4	19.2	2	4	Irregular		48
C	18.5	13.5	17.7	6.5	8,5	Regular		55
D	18	14.3	15.9	2	2,5	Regular		39
E	16	12.7	15.6	1	4,5	Irregular		43
F	17	13.7	15.9	3.5	2,5	Regular		31
G	19	14.7	15.9	3	1,5	Regular		46
H	16	14	15.5	2	4,5	Regular		33
I	17	16.9	17.6	5	2	Irregular		31
J	14	15.2	17.9	1.5	8	Irregular		29
K	17	14.1	16.7	7	4	Regular		30
L	15	13.8	15.2	6	1,5	Irregular*		29
M	18	11.7	13.3	5	2	Regular		47
N	15.5	14.25	16.9	2	10	Regular		41
O	18	14	14.3	3	1	Regular		32
P	15.5	13.7	17.2	2	4	Irregular		24
Mean (Range)	16.5 years (13–19)	14.1 kg/m^2^ (11.7–16.9)	16.5 kg/m^2^ (13.3–19.5)	3.4 years (1–7)	4.2 months (1–10)	Regular N = 10	Irregular N = 6	36 minutes (21–55)

*attended up to 15 sessions, but the activity was prescribed and her attendance irregular.

### Procedure—data collection

Participants (and their parents, for minors) received complete written information about the scope of the research, the identity and affiliation of the researchers, the possibility of withdrawing from the study at any point, and confidentiality. Participants (and their parents, for minors) provided written consent. This research received approval from the institutional review board of the hospital involved, Institut Mutualiste Montsouris, Paris (n°2013–005), and was registered with the French data protection authority (the Commission Nationale de l’Informatique et des Libertés–CNIL) (n°1734833v0).

Data were collected in 16 individual semi-structured face-to-face interviews (by JG) that took place at the IMM hospital, in a consultation room, between January 20 and April 20, 2014. They were audio-recorded and subsequently transcribed verbatim. An interview topic guide was developed in advance and included 9 dimensions with open-ended questions and several prompts (Table 2), constructed to elicit in-depth and detailed accounts of the subject’s feelings during and after the qigong experience, the meaning it had for her, its impact on her body image, and the effects she connected to the qigong practice. Our overall objective was to explore the meaning of the qigong experience for each participant.

Researchers discussed their own feelings about the interviews during study group meetings, to take their potential influences on the data collection and analysis into account (reflexivity).

**Table 2:** Interview topic guide.

Dimensions- Questions and prompts
**Can you describe a qigong session? Possible prompts: What did you do?**
**What was it like to practice in a group? Possible prompts: How did you feel about the presence of other participants? How did you bond to them or not? What about the instructor?**
**How did you feel during the activity? How did you feel afterwards? What did you like the most/the least? Possible prompts: Did you ever find it useful? Did you ever feel bad during or after it? What about your body image?**
**What is the meaning of qigong for you? Possible prompts: what do you think of it? Does it make sense?**
**Do you use any qigong techniques outside of the program? Possible prompts: moves, breathing techniques? How, when?**
**Do you communicate about your experience of qigong? Possible prompts: outside the workshops? With your family? Nurse? Psychiatrist? Other patients? Friends?**
**Compared to the other activities, what is the specificity of qigong for you?**
**Would you like to continue qigong after your hospitalization?**
**Could you please summarize your opinion about qigong?**

### Qigong intervention

Qigong was offered to patients as a part of a multidimensional inpatient treatment program for adolescents with AN [9–11]. This group activity, which started in 2008, was scheduled once a week after lunch (instead of rest time) and lasted for 90 minutes. The instructor was a nurse (JM) with advanced training in qigong (Ling Gui Institute). She conceived the activity as a way to enable inpatients to settle and focus on their body through sensations other than pain or deprivation. She presented the workshop as a way of positioning oneself in space, in movement or at rest, with no demands for performance or results. The following objectives were proposed: rooting/grounding, awareness of one’s central axis, pleasure in body movements, positive reconnection with bodily sensations and feelings, access to interoception. The activity started with a relaxation phase that focused on breathing sensations (10 min), followed by self-massage (20 min) in a seated or lying position, on a thin mattress. Self-massage was designed as a way to connect with and relax the body. It consisted of gentle tapping or rubbing on specific points or paths from the top of the head to the feet. Next, participants performed dynamic qigong exercises, generally at a slow rhythm and with careful attention to breathing (30 min). Examples of forms include: the White Tiger, Wild Goose, Eight Pieces of Brocade, and Jade Body. The instructor adapted the exercises to these young women’s emotions and energy levels. White Tiger, for example, can be used for anger release. Specific attention was given to body placement, laterality, alignment, and body awareness. Patients divided into pairs for another massage phase, where each alternately massaged the other on the head, shoulders, back, and arms with tennis balls (10 minutes for each patient, total of 20 min) in a sitting position. The session ended with a relaxation phase in a supine position intended to focus on inner sensations and feelings (10 minutes).

The qigong activity was tailored to this specific public of female adolescents with AN. Thus, qigong static figures were not used, so that patients could not seek to perform or compete, by attempting to hold the figure as long as possible, with excessive muscle tension. The paired massage phase was added as incentive to participate.

### Data analysis

The qualitative analysis applied the methodology of interpretative phenomenological analysis (IPA), which aims to understand how people make sense of their major life experiences by adopting an ‘‘insider perspective” [27]. Three epistemological points underpin IPA: phenomenology, hermeneutics, and idiography. First, as a phenomenological method, it seeks to explore the informants’ views of the world. As Husserl wrote [28], the objective of phenomenology is to understand how a phenomenon appears in the individual’s conscious experience. Hence, experience is conceived as uniquely perspectival, embodied, and situated [19]. Second, IPA, as defined by Smith & Osborn [29], considers the research activity as an "interpretative activity", because access to the participant's personal world "depends on—and is complicated by the researcher's own conceptions"[29]. It can therefore be described as an hermeneutic approach, relying on an interpretative activity. In IPA there is in fact a double hermeneutic: the ‘‘researcher is trying to make sense of the participants trying to make sense of their world”. Third, the idiographic approach emphasizes a detailed, deep understanding of the individual cases.

The analytic process proceeded through several stages: we began by reading and rereading the entirety of each interview, to familiarize ourselves with the participant’s expressive style and obtain an overall impression. We took initial notes that corresponded to the fundamental units of meaning. At this stage, the notes were descriptive and used the participants’ own words; we paid particular attention to linguistic details, including expressions and metaphors. Next we drafted conceptual/psychological notes, by condensing, comparing, and abstracting the initial notes. Connections with notes were mapped and synthesized, and emergent themes developed. Each interview was separately analyzed in this way and then compared to enable us to cluster themes into superordinate categories. Through this process, the analysis moved through different interpretative levels, from more descriptive stages to more interpretative ones; every concept not supported by data was eliminated. Because of the need to maintain the link between researchers’ conceptual organization and the participants’ words [30], categories of analysis are not worked out in advance, but are derived inductively from the empirical data.

To ensure validity, two researchers (JG and MP, a psychiatrist trained in qualitative research) conducted separate analyses of these interviews and compared them afterwards. A third researcher (NG, adolescent psychiatrist) triangulated the analysis. Every discrepancy was negotiated during study group meetings, and the final organization emerged from the collaborative work of all authors. We did not consider that data saturation was achieved, mainly because we can argue that as many experiences exist as individuals. Nevertheless, we agreed to consider that the data were sufficient once no new aspects emerged from the interviews (i.e., no more codes were added to our codebook) for any of our themes. A sample size of 16 interviews was deemed sufficient. If a minimum of 15 interviews has been suggested for most qualitative research [31], IPA has no formal recommendations (according to studies, sample sizes usually vary from 3 to 15 or more interviews [29]).

We focused on qigong only and do not report in this paper the results related to the massage part of the activity.

## Results

Eleven themes describing the incentives and barriers to adherence to, understanding the meaning of, and the application of qigong were identified and organized into three superordinate themes: individual, relational, and organizational dimensions. The first superordinate theme, the individual dimensions, comprises the attitudes and explanations that the adolescents saw as related to themselves; it includes the themes: (1) attitude towards movement: from hyperactivity to ability to relax, (2) attitude towards a new cultural frame: from Cartesian rationalism to an opening to Eastern philosophy, (3) mind-body attitude: from dualism to integration, and (4) time-related effects. The second superordinate theme, relational dimensions, involved issues with others and were categorized into three subthemes: (5) perceptions of the group, (6) role of the instructor, and (7) family attitude towards qigong. The third superordinate theme covered organizational dimensions, that is, issues related to (8) qigong access policy, (9) setting and degree of compartmentalization of the activity in the overall patient program, (10) focus of the activity, and (11) the activity’s schedule. Within each dimension and even within each theme, we identified drivers and barriers to adherence to qigong, to understanding its meaning, and to applying it outside the workshop. The barriers, in particular, could be specific to the experience of AN or involve the experience of adolescence more generally. Table 3 summarizes the themes and notes the barriers exacerbated by AN. Overall, the participants described a great variety of experiences (S1 File).

**Table 3:** Incentives and barriers to adherence to qigong, to understanding its meaning, and to applying its techniques in other spheres.

Dimensions and themes	Incentives	Barriers
• Individual dimensions ▪ (1) Attitude toward movement ▪ (2) Attitude toward the new cultural frame ▪ (3) Mind-body attitude ▪ (4) Time-related effects	Ability to relax	Hyperactivity ^AN^
	Opening to Eastern philosophy	Cartesian rationality ^AN^
	• Mind-body attitude: integration • Connection to inner sensations and feelings	• Mind-body attitude: dualism ^AN^ • Confrontation with the body ^AN^
	Time-related effects (repetition, recovery)	
• Relational dimensions ▪ (5) Perception of group ▪ (6) Instructor's role ▪ (7) Family attitude	Group conviviality	Fear of the group ^AN^
	Instructor charisma	
	Positive family attitude towards qigong	
• Organizational dimensions ▪ (8) Qigong access policy ▪ (9) Setting, degree of compartmentalization ▪ (10) Scope of the activity ▪ (11) Schedule	Open access	Constraint ^AN^
	Compartmentalization (adherence)	Compartmentalization (application)
	Activity focused on something else than disease	
	Schedule: during rest period	After lunch schedule (postprandial anxiety)^AN^

^AN^ barrier exacerbated in AN.

We develop and illustrate the individual, relational, and organizational dimensions of the participants’ experiences.

### Individual dimensions

Individual dimensions that involved barriers included hyperactivity, Cartesian rationality and a dualist mind-body attitude. On the contrary, the ability to relax, an openness to Eastern philosophy, and mind body integration facilitated adherence, understanding, and application; they were not only incentives to adherence but also its consequences, that is, the effects of qigong.

#### Attitude toward movement: hyperactivity versus ability to relax

Initially, the belief that the activity is about moving can motivate participation. Qigong, however, confronts patients with AN with an aspect of movement they are not used to: its movements are slow and it is associated with the act of letting go. During the activity, patients must deal with slowness and must lie down; these can be barriers. Especially for people with severe hyperactivity, being forced to move slowly or to relax can be experienced as grueling. Patients were frequently unable to elaborate and explain how and why it was so hard to go slow and relax, but they insisted that it was. This difficulty was partially associated with anxiety (especially postprandial), ongoing obsessive thoughts, and the need to face their body. Inability to relax was sometimes associated with a feeling of incompetence: the patient was disappointed at failing to meet the instructor’s directive to relax. This example illustrates how the need to be in control obstructs the ability to "let go" and how perfectionism can lead to negative self-judgment and faultfinding. Other patients were able to relax to the point to falling asleep, but they perceived that as a loss of control and strongly resented it. These issues sometimes even led them to drop out of the activity.

B (irreg): *“It’s true*, *though*, *that when I was very um*… *sick*, *when I was hyperactive*, *I found it was very hard*, *the first class*, *because I couldn’t go slowly*. *And I found it too hard to be asked not to make fast movements*, *to take my time*, *I found that very hard*.*“*

H: *"I wanted to cry because I felt very bad for not having succeeded at*… *relaxing*, *letting go*.*"*

J (irreg): *"The time I fell asleep*, *I really wasn’t doing well (*…*) it made me hate Qigong (*…*) I didn’t want (*…*) I let go and it’s not my thing*.*”*

Some patients, on the contrary, enjoyed the relaxation phase and considered this aspect of qigong an incentive to participation:

A: “*It was interesting because well*, *already it helped me to relax*, *to concentrate on myself*, *to set my breathing to a certain rhythm (*…*) I went there mostly to relax*, *because I really thought it was very pleasant*, *it was relaxing and also let me think about other stuff*, *to let myself go*.*”*

#### Attitude toward the new cultural frame: cartesian rationalism versus openness to eastern philosophy

The participants linked their agreement to go to the Qigong activity specifically to their capacity to accept newness. Curiosity and an open mind led them to try it, and those who reported an appetite for esoteric or incomprehensible practices found adherence easiest:

G: *“It seems yeah*, *well*, *I don’t know*, *yeah I don’t know*.... *It’s not that I believe in everything but in general I am pretty open to different activities that*, *that are sometimes very irrational so um*… *it’s true that Qigong*, *I went to it*, *and um*… *I said to myself ‘Sure*, *why not’*.*”*

On the other hand, rationalism appeared to be an obstacle to adherence. Several young women reported finding it difficult to accept the metaphoric meaning of qigong body movements. They explained that their problems in making sense of these movements led them to be skeptical about the usefulness of the practice and prevented full adherence:

E (irreg): *“Sometimes she would say stuff like ‘the goose is protecting itself from danger’ or I don’t know what these were (gestures) where we would ask ourselves*… *what does that mean (laughs)*… *well I thought the gestures were a bit weird*.*”*

C: *“Because I think that there’s a part of*, *well*… *yes there’s a part of belief*… *Qigong you have to*… *it’s a little like*, *well*… *not a religion*, *but there’s something where you have to believe in the energies*, *the fact that*, *and so*… *well we get used to something very rational etc*… *And that can be a little disturbing (laughs)*.*”*

Moreover, the arcane aspects of qigong could even be experienced as implying that they should put their ego aside and question their self-esteem.

C:*”It involved letting go a little bit and*… *well*.... *put aside all personal esteem*, *it’s a little … a little unsettling at the beginning*, *um*, *finally it’s more like a game really*, *but it was fun*, *well I liked it*.*”*

Not liking letting go may be related to the need for control and perfectionism frequently met in patients with AN. Gamification of the activity can be a necessary step to allow oneself to experiment with qigong and experience it. It lets the patient go around the obstacle. In the example, once the patient let herself experience the qigong, she reported that she enjoyed it.

#### Mind body attitude: dualism or integration

In qigong, the patients are asked to focus on their body sensations. Depending on how the patient integrates her internal perceptions, qigong practice can lead to either constructive body awareness or to a difficult confrontation with a hostile body image. Some patients even reported that being forced to face their physical body was associated with fear of relapse. This was particularly salient when patients were gaining weight.

B (irreg): *“I think that um*…*at times*, *well if we feel our bones a little less*, *precisely if we don’t feel we have muscles anymore*, *that we have flesh*, *all that*, *I think that it can make you*, *sometimes*, *relapse*.*”*

On the other hand, in an integrative perspective, qigong was experienced as a way to reconnect with inner sensations and feelings and to allow mind-body integration—as a tool for helping them to recognize and regulate emotions.

H: *“It lets us listen better*, *well*, *listen to our feelings*.*”*

I (irreg):”*When I’m angry*, *I do the tiger and when I am very tense*, *I do the wild goose*.*”*

Some patients even reported they resorted to qigong during psychotherapy.

B (irreg): *“When we rubbed our feet*, *well the balls of the feet and then*, *it really did anchor into the ground*, *it’s*… *I found that was uh*… *well it really worked to anchor myself in the ground*. *(*…*) it was kind of like if I had roots (*…*) for sessions*, *it helps (laughs*…*) because for sessions I really need to say hard stuff*, *that I don’t necessarily want to say and I’m frequently tempted to escape and all and this allows me to stay anyway*… *and to succeed in getting it out um*… *not all at once but small parts at least*.*”*

This process could lead patients to consider their body differently, as a potential source of comfort.

M: *“We were saying to ourselves that in the end a body can also live and bring a little of (feeling good)*… *so it’s not always a weight to carry*.*”*

For some, qigong echoed prior practices of dancing (or similar activities) that promoted interoception and the experience of mind-body integration. Patients often compared their experience of qigong with their experience of dancing.

M: "So *even though I had stopped before I was in the hospital because um*… *I didn’t find pleasure in it anymore … just being in my leotard*… *making my body talk*, *well it wasn’t good anymore*, *even though with qigong it’s come back*.*”*

#### Time-related effects

Spending time on the activity and doing it repeatedly, or even just letting time pass was sometimes necessary to enable these young women to adhere to and benefit from the qigong. Moreover, patients with previous hospitalizations sometimes experienced qigong very differently the second time, probably due to progress in their recovery. For example, in the overall treatment process, they may have worked on their perfectionism or on their need to be in control. Thus, earlier obstacles to qigong practice may have been overcome, so that the patient is able to experience new aspects—such as relaxation or interoception—and potentially benefit from it.

D: *but after*… *I started to see that*… *each time I came back from qigong I was*… *more relaxed*, *less tense*, *and I started to tell myself that*… *it works*.*”*

H: *"it had to be perfect*, *so it wasn’t relaxing me*, *it was irritating*, *well*… *I know that last year I went to qigong twice and I came out and I was irritated (*…*) and now finally I can do what I want*. *So now it relaxes me more*.*”*

While a long period of time was sometimes necessary to allow adherence, the perceived effects of qigong appeared, on the contrary, to be very time-bound, at least according to the patients’ subjective representations. These effects were either be felt at the moment or over a very short period. Lasting effects were not reported. Some patients reported considering continuing qigong after discharge, but none of these plans panned out, at least as far as we know.

A: *"For me it’s feeling on the spot*, *at the moment*, *the activity was good for me*, *I wouldn’t say it had an influence after*.*"*

I (irreg):*"Well*, *for example I never have a snack*. *After qigong*, *I did have snacks because I felt good!"*

### Relational dimensions

The three themes related to relational dimensions mostly involved incentives to adherence: a positive perception of the group—except when the participant feared the group, instructor charisma, and a positive family attitude.

#### Perception of the group: conviviality versus fear

The qigong workshop was a group activity. This dimension could be experienced as an incentive but also as a barrier. Some participants mentioned their fear and insecurity about being with others. In that case, the presence of another was perceived as potentially intrusive, as persecutory rather than supportive. The negative feelings reported by some patients could also be due to the patient's own perception of the others, including comparison and self-deprecation.

H: “*It made me uncomfortable that there were other people (*…*) I was looking at them for how they were compared to me*, *mainly … when they were thinner*, *that made me feel bad*.*"*

On the other hand, group practice can encourage patients to participate the first time, to adhere, and to practice outside of the workshop. Some patients reported having fun practicing together in the hospitalization unit on different occasions. Beyond conviviality, the group practice sometimes gave them a strong and satisfying feeling of connection and sharing. Group membership was also sometimes reassuring. Participants allowed themselves to experiment with qigong moves they would not have considered doing alone.

B (irreg) *“The fact that we’re in a group and*, *well*, *everyone is doing the movements*, *in fact*, *that created a kind of group harmony*.*”*

D: “*We don’t feel alone doing stuff*. *Because sometimes you seem stupid doing certain movements and there*, *exactly*, *it’s not*, *because it’s in a group so it’s more natural*.*”*

#### Instructor's role

The patients unanimously acknowledged the key role of the qigong instructor. Her charisma was an important incentive to participate. Her investment and her attitude made her a role model for the patients. Her strong charisma, on the other hand, could be considered as both an incentive to participation but also a barrier to continuation of qigong after discharge, for some patients could not imagine practicing this activity with another instructor.

G: *“What was good in qigong was first of all Jocelyne (laughs)*… *It was*, *it was*, *well*… *well she made us want to come*.

K: *"She really listened to us and um*… *was good at what she did and that made us want to do it correctly and feel what she wanted us to feel*.*"*

M: “*Actually*, *I would have liked to continue but for me qigong was Jocelyne and not with someone else*.*"*

#### Family attitude

Their family’s positive attitude towards qigong also lent credibility and attraction to the activity and encouraged patients to adhere.

M: “*Well*… *my mother um*… *she had talked to me about it and in fact my parents had already found out about qigong when I was little for my brother (*…*) um*… *so I took it as a real activity and*… *something serious*.*”*

### Organizational dimensions

Organizational dimensions included four themes: the activity access policy (open access versus constraint), setting (degree of compartmentalization of the activity in the overall program), scope of the activity, and schedule.

#### Activity access policy: constraint versus freedom

As mentioned before, the qigong program was prescribed for only one of the 16 patients, and she did not adhere to it and tried to find ways not to participate. None of the others felt that the activity had been prescribed to them, even if it might have been strongly recommended by the staff. The patients welcomed this open-access policy. Nevertheless, a delicate balance must be found to ensure they try out the activity, because attendance can lead to experimentation with it, and experimentation to understanding and adherence.

P (irreg and prescribed): *"Well*, *I went there as little as possible (*…*) either I would say ‘no’ or I would go somewhere else*, *or I would say that I was going to another workshop*, *or something like that*.*”*

L (irreg) *"and given that I was there and I did it*, *and then after*, *I saw*, *I sort of understood how it worked*.*”*

The workshop itself was considered quite flexible. Nevertheless, some experienced constraint in imposed moves and were ambivalent toward it: they disliked feeling constrained or required to do something, but acknowledged the potential benefit of experimentation with something new that could lead to learning and progress.

#### Setting—degree of compartmentalization

A form of geographical compartmentalization was implemented as the activity was generally offered outside the hospitalization unit (on a different floor, except when a meeting was scheduled in the room). This form of compartmentalization promoted patients' sense of freedom and their personal investment and they enjoyed it.

O: "*Well um*… *I don’t know*, *qigong was*… *was on the first floor*, *well*, *not in the department*.

Investigator: *“And if it was in the department*, *what difference would that have made to you?*

O: *“Well I think it would have been more complicated to really care about it so much*..*"*

Moreover, patients experienced this activity as clearly separate from the rest of the global management program, and they did not talk much about it with either the nursing or medical staff.

#### An activity not focused on AN

Patients appreciated that the activity, like many of the group activities in our program, was not focused on their disease. Instead, it promoted involvement and activity.

F: “*to not think and not see our problems*, *be in another universe*, *a moment that we’re not being talked to about*… *everything you talk to us about here*. *Yes*, *it’s nice*. *Because for once*, *we are told ‘Exactly*, *forget it! Don’t worry about that*, *you have 90 minutes just for yourself*, *keep it for you!’”*

#### Time schedule

The workshop was scheduled after lunch, at a moment of postprandial anxiety when the principal alternative activity was quiet time in their room. This was an incentive to participate, as patients were happy to be diverted from their anxiety and to avoid rest time. It was also however a barrier to full involvement in the activity, because the patients felt especially uncomfortable at this moment. In practice, avoiding rest time was one of the main incentives reported for participation. Although some patients reported difficulties in becoming involved due to the scheduling, the timing was more generally considered adequate because it provided the patients with a solution to their rest-time distress, at least once a week.

B (irreg): “*For me it was more to fill in the gaps and not be depressed in my room*.*”*

I (irreg): *" Well*, *it was good because it was after the meal*. *So it was a time when we were all tense in general so um*… *it was good to be able to be the tiger or the wild goose after that*.*”*

**Fig 1:**
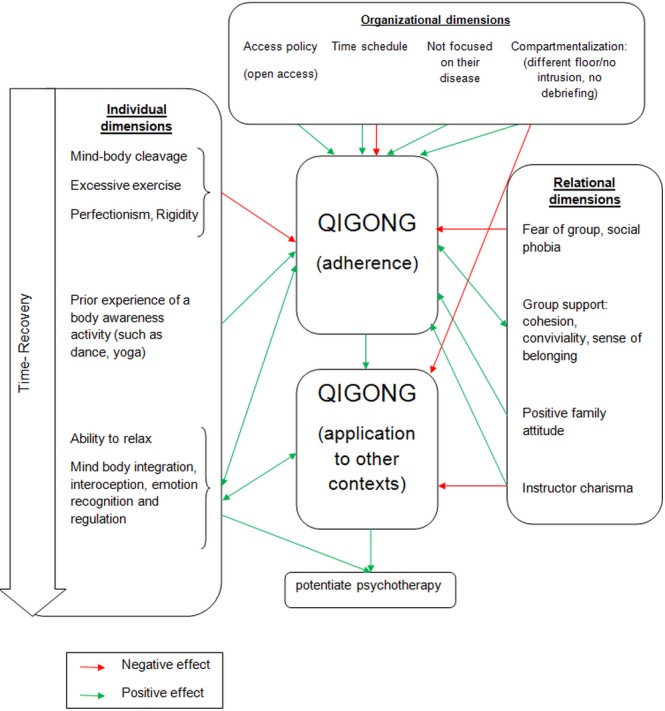
Summary of main incentives and barriers to adherence and application of qigong.

Fig 1 summarizes the different dimensions and their effect on qigong adherence and its application to other contexts.

## Discussion

Our study aimed to (I) investigate the experience of qigong from the perspectives of adolescents practicing it as part of an multidisciplinary inpatient treatment program; and (ii) describe the incentives and barriers to adherence to qigong, to understanding its meaning, and to its application outside the program. The experience of our participants was described by 11 main themes, belonging to 3 superordinate themes or dimensions: individual, relational, and organizational.

- **The individual dimensions** identified in the participants’ accounts—attitudes toward movement, toward the mind (and especially towards rationalism and Eastern philosophies), body-mind integration, and the passage of time—are all factors that help to explain patients’ level of adherence and the meaning they attribute to the activity. Their varying degrees of hyperactivity and different perceptions and conceptions of the interrelations and interactions of mind and body all played an important role. A strong mind-body cleavage and especially excess physical exercise are major features of AN that tend to be barriers to initial adherence. Excess exercise is characteristic of up to 80% of patients with AN [32] and includes a strong drive to exercise and improved muscle tone as a priority reason for exercise [33]. These points explain why patients find it difficult to cope with the slowness/stillness and relaxation of qigong. Hyperactivity can be both a weight-loss strategy and a kind of compulsion or even addictive behavior [34]. Obsessive thoughts too can be part of AN and can, as shown above, make the process of "letting go" really challenging.

Experiential avoidance, perfectionism, and lack of flexibility—all widespread in patients with AN [35–37]—are described as factors involved in the maintenance of AN [38] and even associated with poor outcome [39]. Qigong addresses these issues by inviting participants to participate in a non-competitive, non-Cartesian activity and to experiment with different internal experiences. Qigong confronts young women with questions about body awareness, emotions (recognition and regulation) and even spirituality. From the patient’s perspective, these are all known to be key factors in the recovery process, which includes self-acceptance, determination, and spirituality as equally important elements [40]. In a dualist attitude, mind and body are considered as two separate entities [41]. The body is perceived as “muscles and flesh” with no connection with the mind. In a phenomenological approach, we can distinguish the physical or object body (German: *Körper*) from the lived or subject body (German: *Leib*) [42]. In eating disorders, effects at the level of the physical body may disturb embodiment related to the body image [42,43]. Qigong invites people to focus on both physical body sensations and subject body experience and can thus lead to what is also called “mind-body integration", that is, an embodied sense of self. Focus on the object body can, however, reactivate the disturbance of some patients’ embodiment and force them to face an unpleasing body image. Awareness of this risk is essential for clinicians to detect and manage it on an individualized basis for each patient.

Interoceptive abilities are known to be affected in eating disorders [18]. Interoceptive awareness assessed by the eating disorder inventory-2 (or 3) is reduced in BN [44] and in ED in general [45], whereas interoceptive accuracy, assessed by the heartbeat detection task, was only reported to be reduced in AN [19]. Interoceptive accuracy has been reported to be negatively correlated with self-objectivation, i.e. the tendency to experience one’s body principally as an object. People with lower interoceptive accuracy tend to evaluate their body for its appearance rather than for its effectiveness [46]. Moreover, interoceptive accuracy is considered as *“a potential independent and stable factor of AN*,*(…) that is not touched by state-of-the-art cognitive behavioral therapies”* (see p.6 in [18]). Mindfulness techniques and interventions using interoception as a core element, such as qigong, could therefore be of great interest to potentiate interoceptive accuracy and improve interoceptive processes [44]. Difficulties in perceiving one’s own internal sensations may well be associated with problems in recognizing and paying attention to emotions, which manifest themselves both physically [47] and mentally. Recognition and regulation of emotions are known to be impaired in AN [48], and alexithymia is associated with the persistence of AN [49]. Benefits of qigong, as reported by some patients in our study, include access to interoception, "the process of receiving, accessing and appraising internal bodily signals" [50] and to emotion regulation. Awareness of emotional states is a prerequisite to emotion regulation, which implies another prerequisite: interoceptive awareness, that is, awareness of bodily signals. It appears crucial for regulating the intensity of emotional experience and for higher order processing of emotional stimuli [42]. In particular, interoceptive awareness can facilitate successful emotion regulation through reappraisal [51]. Qigong practice could thus help to identify and regulate emotions through enhanced interoceptive awareness. This could be of great interest both in AN and BN, as both share reduced interoceptive awareness, as measured by the Eating-disorder-inventory-3, that more specifically investigates interoceptive awareness of emotional states [45]. These benefits in terms of interoceptive awareness and emotion regulation could in turn potentiate psychotherapy.

To summarize, individual dimensions frequently observed in patients with AN can present serious obstacles to qigong adherence. Once these are overcome, however, patients do report positive effects: satisfaction associated with relaxation and with the experience of mind-body integration. Accordingly, an essential question is whether individual characteristics (such as excessive exercise and mind-body cleavage) should be considered as barriers to qigong practice or should be overcome by this practice. The answer lays probably partially in figuring out the right time to introduce qigong to each patient.

Certainly, time issues seem major: if initiated too early, patients may be less prone to adhere and in some cases, even subject to additional difficulties. Priority for patients with severe AN must go to renutrition [9], which must precede activities such as psychotherapy and qigong. After that, qigong and other body awareness therapies can serve as a first step in erasing mind-body divisions and thereby potentiate psychotherapy. The right moment to introduce qigong must be determined individually for each patient, for their incentives and barriers to qigong adherence differ, as does their readiness to change. Motivation to change, which is an important predictor of outcome in AN, can be worked on and can increase during hospitalization [52]. We suggest it can also affect adherence to qigong and helps explain why it increases over time. Multidimensional programs require patients to deal with many healthcare providers and involve different coordination and communication schemes among the professionals. We think it is important that the psychiatrist in charge assess the motivational aspects and the risk-benefit balance of the qigong program for each patient. Individualizing the treatment approach to each specific patient is of key importance for effective outcomes [53] and may be facilitated by a debriefing with a psychiatrist after the first session to distinguish patients who can benefit from qigong from patients for whom it should be postponed and to decide how to best support each patient. Some patients could then be advised to try at least 3 sessions before another debriefing.

- **Relational dimensions**, that is, how patients perceive the group (as a threat or support), family attitudes about qigong, and the relationship with the instructor, also help explain level of adherence to and ability to find meaning in qigong. This dimension appears predominant in the adherence process.

Because most patients consider the group supportive, group qigong practice seems appropriate to them, even though the negative body image in AN [54] and frequent social phobia [55] can reinforce fears of judgment by their peers. These fears, moreover, are valid: females with AN estimate other women's weight higher than healthy women do and consider extremely underweight women to be more attractive and normal- and overweight women less attractive [56].

The instructor is considered a role model and an inspiring figure. Research has shown that the therapist matters and is indeed the most important contextual factor determining the effectiveness of complementary and alternative medicine [57]. Our results confirm the primary importance of the provider. A better understanding of human effects in any therapeutic mediation is still needed.

Patients reported that a positive family attitude promoted adherence, consistent with studies showing that parental motivation and parent-therapist alliance predict positive child outcome in a variety of treatments for indications ranging from mental health to diabetes [58,59]. Moreover, the parents’ and children’s alliances with the therapist may interact synergistically: it has been suggested that the parental alliance may promote treatment completion, while the child’s therapeutic alliance affects treatment outcome [60]. More generally, family involvement is important in AN management. For example, the addition of family therapy focused specifically on intrafamily dynamics to an established integrative multidisciplinary outpatient treatment program for adolescents with AN significantly improved outcome at 18 months of follow-up [61].

In summary, we see that most aspects of the relational dimensions appeared to serve as incentives to participate and helped make the activity meaningful, through conviviality, a sense of belonging, and sharing. Motivation thus relies mostly on these relational dimensions, whereas the individual dimensions played a much more mixed role in adherence. This point may also explain the very limited continuation of qigong after hospitalization: the group- and instructor-related motivating factors are no longer present.

- **Organizational dimensions.** Access policy, time schedule, scope of the activity, and the integration of the qigong activity in the multidimensional treatment program are important motivation factors. Different adjustments and settings may have important consequences on adherence to and continuation of qigong. In our study, these dimensions tended to increase qigong adherence, but also to limit its post-discharge continuation.

First, the balance between freedom of access and constraint merits discussion. Constraint may be necessary to compel patients to experiment [62] but freedom, or at least a trusting relationship, is also needed to promote adherence [63]. A qualitative study reports that young women with AN agree that compulsory treatment is justified in life-threatening conditions but not otherwise. Most important to them, however, was not the restriction of freedom but the nature of their relationships with the professionals, for "within a trusting relationship compulsion may be experienced as care" [63]. As the NICE guidelines state, "a precondition for any successful psychological treatment is the effective engagement of the patient in the treatment plan. Health care professionals involved in the treatment of AN should take time to build an empathic, supportive and collaborative relationship with patients and, if applicable, their carers" [64].

Second, the timing of the program, here after lunch, can be especially difficult for some patients. Patients may be prone to postprandial anxiety and want an active treatment to deal with it [65]. Moreover, as mentioned above, the workshop was scheduled during a rest period that many patients wanted to avoid; patients with AN perceive bed rest negatively, as a source of isolation and boredom [66]. A different timing (before dinner or before bedtime) might alleviate the disadvantages associated with the postprandial schedule. Ideally patients could start with an evening program and then add or switch to the after-lunch program.

Third, the degree of both compartmentalization and integration in the overall treatment program should be discussed: compartmentalization promoted access to the activity in the first place, simply by offering an activity not focused on AN. This focus on quality time unrelated to their disease appeared to be an incentive to adherence here, as reported in previous studies [67]. Nevertheless, it remains an activity focused on the body, and ambivalence is present: focus on the body can arouse interest and slowly lead to another perspective on the body, but it also risks exposure to the body and increases negative emotions [68]. Integration and involvement of the care team can potentiate the scope of the activity, encourage the application of qigong techniques, and increase patients' involvement and empowerment. Other studies have stressed the importance of staff involvement in activities for people with psychiatric disabilities [69]. However, the borderline between involvement and intrusion is a fine one. Different forms of organization could be considered. For example, a team member besides the instructor could attend the workshop. This would make linkage and communication with the staff easier and avoid regular direct patient interviews that might be perceived as intrusive. A scheduled discussion period could be routinely proposed to participants at the end of the activity on a given schedule (every session, every two sessions, once a month…).

Taking ownership of qigong and continuing it outside the institution appeared limited in our study sample. This may be partly explained by the prominent role of the instructor: the activity is personified for these patients, so that they find it difficult to experiment with qigong with someone else. More generally, this is probably related to issue of motivation, discussed above. Motivation in this case depended mostly on the social and relational benefits of the group activity, i.e., extrinsic factors. Strategies involving those extrinsic factors could be used to encourage continuation of qigong, such as allowing patients to continue the program at the hospital after discharge, providing them with the benefits of the group. This could offer a continuum and a successful transition. Referral to another qigong provider by the instructor might also reassure the patients and provide a sort of launching pad. Still, some general questions remain, including the type of group (therapeutic or not?) to which they should be referred, and the financial aspects, which may be a barrier for some. Beyond those points, however, consistent continuation of qigong after discharge probably requires intrinsic motivation factors. Serious consideration of how to support patients in finding autonomous motivation would be beneficial. Certainly, self-determination theory suggests that autonomous (i.e., intrinsic and identified) forms of motivation are much more effective than controlled (i.e., introjected and externally regulated) forms [70].

### Strengths and limitations

The generalizability of these results, like those of any qualitative study, is limited. Here, this is related to both the intervention and the public. First, the qigong intervention was tailored to the specific public in our program: adolescents hospitalized for AN. Second, although our purposive sampling allowed us to include a wide range of experiences among female adolescents who did and did not adhere to the qigong intervention, our findings can only be generalized to young women hospitalized and exposed to similar interventions and treatments.

As mentioned in the results, patients had difficulty elaborating on some of their thoughts and feelings in the interviews. This may be related to the association between low BMI and cognitive impairment [71].

Nevertheless, to our knowledge this is the first qualitative study exploring the experience of qigong among adolescent patients with severe AN. It has helped to identify incentives, barriers, and perceived effects and risks that may be encountered in other mind-body therapies. It also raises questions about time issues and the activity’s place in overall management that qigong shares with other body awareness therapies.

Last but not least, we showed the utility of qualitative studies in considering the individualization of treatment approaches, as they allow a profound understanding of the differences between patients that quantitative studies do not usually provide. For example, this study noted that the same components of qigong were experienced as positive and helpful by some patients and as a barrier or hardship or even a danger by others. We also pointed out that for patients to benefit from qigong, the right moment to introduce it must be determined individually.

## Conclusions

Qigong appears to be an interesting therapeutic tool that can potentiate psychotherapy and contribute to the recovery process in patients with AN. Individual dimensions associated with AN may curb adherence, whereas relational dimensions appear to provide incentives to join the activity. Further analysis of the best time window for initiating qigong and of its place in overall management might help to overcome some of these barriers, limit the risks, and maximize its benefits. Systematic debriefing with a psychiatrist after 1 to 3 sessions should be considered, and scheduled time to discuss the activity could be included in the workshop.

More globally, the development and evaluation of mind body activities (such as qigong) for patients with eating disorders appears as an asset. Such activities could lead patients to perceive their body in a positive way, counterbalancing the negative experience of impaired body image and food associated uncomfortable body sensations existing in eating disorders, and ultimately allow them to access to an enhanced sense of self. Moreover, it could lead patients to reconsider their perception of stillness and rest and allow them to access to relaxation and benefit from it. Taken together, increased relaxation, reduced body-related anxiety and better body acceptance could promote the adhesion to the renutrition program.

Future research dedicated to assessing the impact of qigong and other mind-body interventions on bodily signals perception, body acceptance, emotional awareness and emotion regulation would be of great interest.

## Supporting Information

S1 File Theme-related quotations from interviews. (PDF)
