# Supplementary material for: Group Qigong for Adolescent Inpatients with Anorexia Nervosa: Incentives and Barriers
Source: PLoS One. 2017 Feb 2;12(2):e0170885. doi: 10.1371/journal.pone.0170885 (PMC5289494; doi:10.1371/journal.pone.0170885)
Supplement: S1 File — (PDF) [file pone.0170885.s001.pdf]

Supporting Information: Theme-related quotations from interviews

Theme	Quotation from interview
(1) Attitude toward movement	. The belief that the activity is about moving is an initial motivation to participate<br>*"Well I, I didn't really know what to do. I saw it on my schedule. I didn't know what it was. The others told me it was a few... some movements so when I heard the word movement, um... I went."*<br><br>. Inability to relax<br>E (irreg): *"I didn't really like the relaxation*. B*ecause I couldn't make myself relax, well... I wasn't into it, I... well, it wasn't very...I didn't like it (...) that's what was complicated, to put aside... my thoughts, all that and... concentrate on my body. It was hard! »*<br><br>. Having to lie down can be an obstacle<br>L (irreg): *"And then finally when I saw that at the beginning we were supposed to lie down on the mats, that slowed me down a little."*<br><br>. Those difficulties could lead patients to drop out of the activity.<br>L (irreg): "*I was counting the time, and then I was looking at the time every minute, to see, when am I going to stand up again (…) In the end, it's not that I didn't like it at all, but it was too complicated and I ended up not going any more. »*
(2) Attitude toward the new cultural frame	. Curiosity leads patients to test the activity<br>M: "*because it was intriguing, it was really out of curiosity. And um... Yeah, it was curiosity! And then finally, I liked it."*
(3) Mind-body attitude	. Qigong can make patients have to confront their body that is gaining weight.<br>C: "*But after, I found that for anorexia and all, it's not necessarily what's best! Because the fact of feeling your body when you are gaining weight back (…) that can be super-complicated. So I don't think that it (qigong) is the best thing for anorexia."*<br>. Qigong as a tool of mind-body integration<br>K: "*It's a moment for yourself and a moment of being related to your body. Finally, good, we listen a little to our bodies."*<br>C: "*Finally… the body and the soul basically, they go together, in fact…"*
(4) Time-related effects	. Long-term effects were not reported<br>O: *"Afterwards, I don't know exactly if it gave me something in the long term. I don't think that in the long term it changed much."*<br>. Continuation of qigong was considered but did not work out<br>G: *"In fact, we think that here because it's the framework that's like that. It's one of the workshops, is the thing! But yes, outside uh... well, at the beginning, I said to myself, 'Yes why not try it outside.' And then you're out, you take your life up again, and everything, and uh... it just went completely out of my head."*
(5) Perception of the group	. Group practice encouraged patients to participate<br>N: "*Most of the people liked it and so, uh... I was seeing lots of people*

	who were going too and that made me want to discover what it was."<br>.Feeling of connection and sharing<br>N: "*Yes, it's true that …mmm… you felt that the room was full, uh... that it was really nice to be able to share an activity with people you like, share an activity that you like with people you like, that's always better than all alone."*<br>. Esthetic considerations are even reported:<br>D: *"I think that when a movement is done well, there's an overall effect that must be really pretty."*
(6) Instructor's role	. Key role of the provider - ability to find the right attitude<br>N:*"the instructor is, she, uh ... finally the main thing about qigong, it was a really really good teacher."*<br>I (irreg): *" the fact that she put herself at the same level as us (...) We had the impression that she really wasn't one of the nurses, that she was doing qigong like us... like us patients.(...) she was gentle and at the same time firm. (...) An iron hand in a velvet glove! (...) Yes! Because at the same time she directed us exactly and she did it so gently that we didn't feel alarmed at all."*<br><br>. The instructor can be so important that patients will not practice qigong outside the hospital.<br>G: *"Well already in general when I applied things from qigong, it was always here. That is, outside, at home, finally I wasn't thinking about qigong at all anymore, so ... that was it."*
(7) Family attitude	. Family support<br>N*: "Yes, yes, to my family, yes I talked about it and yes, finally, they were interested, they knew about it already and uh... so I talked to them about it a little. They wanted me to show them and tell them what we did."*
(8) Qigong access policy/Attitude toward constraint	. Ambivalence toward imposed moves<br>B (irreg): *"Qigong, it was more compulsory figures. Well, I'm not saying that it was bad to make us do compulsory figures, but it was different from improvisation, really.*<br>***Investigator: Mmm. And what does it mean that they were compulsory?***<br>*Well, sometimes some of us didn't want to do them. Me, for example, the water figure, I didn't want to do that, I know that I didn't want to do it.*<br>***Investigator: Mmm. So it was more of a constraint, you would say? The fact that it was compulsory.***<br>*Well, no, yes and no… Because… No because if we never did it, we wouldn't learn much. And yes because if there were figures you didn't want to do or that you didn't like, well, you still have to do them."*
(9) Setting (degree of compartmentalization)	. Patients did not share their experience of qigong with the nursing or medical staff<br>*Investigator: And did you talk with the nurses or the doctors about qigong, about what you were doing, is that …?*

	N*: Um... no!*<br>*Investigator: Would you have liked to talk to them about it?*<br>N*: (Silence)… Well, no, I'd prefer that to stay in the framework of qigong, of an activity here, really.”*
(10) Scope of the activity (not focused on AN)	. The activity promotes an active role<br>C: "*I think that as therapy it has a sort of optimistic side, where you have the impression of being active in something, in fact."*
(11)Time-schedule	. Avoiding rest time<br>F: *"Because upstairs, they make us rest after lunch until 3 pm. It's interminable."*<br><br>. A period of postprandial anxiety that can prevent full involvement in the activity<br>A: *"It was right after lunch, and when I got there it was still pretty tense, we don't always feel so well after a meal."*<br><br>B (irreg): *"Well, that's what was hard the first few times because exactly, it was after lunch. Afterwards, it was ok, but yes, it's true that sometimes I still had the impression that it was keeping me from digesting my food. That it was sitting on my stomach."*<br><br>. Some patients especially like the after-lunch schedule<br>N: *"Me, I thought it was really ... uh, the best time it could possibly have been because uh... it was ... a little after lunch."*

(irreg): indicates that the patient had a history of irregular attendance.

AN: Anorexia Nervosa
